# Reforming Taiwan’s Living Kidney Donation Policy: Achieving Consistency and Healthcare Equity

**DOI:** 10.1016/j.ekir.2026.106531

**Published:** 2026-04-02

**Authors:** Hung-Bin Tsai, Chin-Hui Pan, Feng-Jung Yang, Ming-Che Lee

**Affiliations:** 1 Taiwan Organ Sharing Registry and Patient Autonomy Promotion Center (TOSRPAPC), Taipei, Taiwan; 2 Division of Nephrology, Department of Internal Medicine, National Taiwan University Hospital, Taipei, Taiwan; 3 School of Medicine, College of Medicine, National Taiwan University, Taipei, Taiwan; 4 Division of Hospital Medicine, Taipei City Hospital-Zhongxing Branch, Taipei, Taiwan; 5 Department of Medical Genetics, National Taiwan University Hospital, Taipei, Taiwan; 6 Division of General Surgery, Department of Surgery, Shuang Ho Hospital, Taipei Medical University, New Taipei City, Taiwan; 7 Department of Surgery, School of Medicine, Taipei Medical University, Taipei, Taiwan; 8 Research Center for Organ Transplantation, College of Medicine, Taipei Medical University, Taipei, Taiwan

### Introduction

Taiwan faces a critical organ shortage; over 8500 patients await kidney transplantation, whereas approximately only 430 transplants are performed annually.^1^ Living donor transplantation offers superior outcomes—higher graft survival, better renal function, and improved quality of life^2^—yet, access is severely constrained by an unjustifiable regulatory disparity. Although living liver donation permits donors from blood relatives within 5 degrees of kinship and relatives by marriage aged ≥ 18 years, kidney donation remains restricted to blood relatives and spouses only, aged ≥ 20. This inconsistency lacks medical or ethical justification.

### The Regulatory Inconsistency

Taiwan’s Human Organ Transplantation Act, enacted in 1987, has undergone 4 amendments progressively liberalizing liver donation, while maintaining restrictive kidney donation standards (Tables 1 and 2). The 2021 amendments expanded liver donor eligibility to include relatives by marriage and reduced minimum age to 18, aligning with the civil law. Kidney donation remained unchanged—paradoxically, despite kidney donation carrying substantially lower surgical risk than partial hepatectomy.^3^

**Table 1:** Taiwan's current regulatory framework

Criterion	Liver donation	Kidney donation
Blood relatives	≤ 5th degree	≤ 5th degree
In-laws (by marriage)	✓ Permitted (≤ 5th degree)	✗ Not permitted
Spouse	✓ Permitted	✓ Permitted
Minimum donor age	18 yrs	20 yrs

**Table 2:** International comparison of living kidney donation (2024 data^a^)

Country	Deceased kidney, n	Living kidney, n	Living rate (pmp)
United States	22,076	6418	18.78
United Kingdom	2299	943	13.87
Germany	1443	632	7.59
Japan	233	1544	12.52
Taiwan^b^	240	191	8.16

pmp, per million population.

a Source: International Registry in Organ Donation and Transplantation, 2024.

b Despite having 313 living liver donations in 2024, Taiwan's living kidney donation rate significantly lags behind comparable developed nations.

### Historical Context: Why Liver First?

The liberalization of living liver donation in Taiwan preceded kidney donation reform because of 4 converging factors. First, fewer deceased donor livers are available, because cultural resistance to brain death determination and cadaveric organ donation—deeply rooted in East Asian Confucian and Buddhist values—severely constrains the deceased donor pool. Unlike kidneys, where each deceased donor yields 2 organs, a usable deceased donor liver is far scarcer, creating a supply deficit that advocacy groups could leverage directly. Second, Taiwan’s historically high prevalence of chronic hepatitis B infection—exceeding 15% of the general population before universal vaccination—produced an exceptional burden of cirrhosis and hepatocellular carcinoma, generating sustained patient advocacy and policymaker attention to liver disease that had no parallel in nephrology for decades. Third, end-stage liver failure has no adequate bridge therapy; unlike kidney failure patients who survive on dialysis for years, patients with terminal liver failure face imminent death without transplantation, giving policymakers an unambiguous humanitarian imperative unavailable in the kidney setting. In contrast, Taiwan’s exemplary renal replacement therapy infrastructure—consistently ranked among the world’s highest in dialysis access and quality—meant that kidney failure was perceived as manageable, reducing the political urgency for transplant policy reform; this perception, though understandable as a contextual factor, does not negate the well-documented survival benefit and quality-of-life advantage of transplantation over long-term dialysis. Fourth, and biologically fundamental, the liver regenerates after partial hepatectomy, recovering 80% to 90% of the original volume within 6 months, whereas the kidney has no regenerative capacity; unilateral nephrectomy results in permanent loss of approximately 25% to 30% of total nephron mass. This biological distinction, combined with Taiwan’s world-leading liver transplant outcomes at centers such as National Taiwan University Hospital and Chang Gung Memorial Hospital, allowed policymakers to expand liver donor eligibility with demonstrated evidence of donor safety. No equivalent safety evidence base had accumulated for expanded kidney donation, partly because the policy environment had not created a demand for it. Notably, Taiwan’s own Taiwan Organ Sharing Registry and Patient Autonomy Promotion Center convened an expert consultation in May 2020 specifically to evaluate liberalizing living donation kinship restrictions; the meeting concluded that harmonizing kidney donation standards with liver standards and removing spousal eligibility conditions was appropriate, and formally recommended that the Ministry of Health and Welfare initiate legislative amendment. This official consensus has yet to translate into enacted reform.

This disparity particularly disadvantages patients with hereditary kidney diseases— autosomal dominant polycystic kidney disease , Fabry disease, and other genetic nephropathies—whose blood relatives may themselves be affected or at-risk. In cases where only blood relatives can donate, many such patients face indefinite dialysis. The Taiwan Polycystic Kidney Disease Patient Association has advocated that extending eligibility to relatives by marriage would substantially expand potential donors for these patients.^4^

### Quantifying the Impact

The policy gap creates an estimable, if conservative, burden of foregone transplants. Autosomal dominant polycystic kidney disease —the most common hereditary kidney disease, with a prevalence of 1:400 to 1:1000—accounts for approximately 5% to 10% of end stage renal disease cases globally; applied to Taiwan’s 8500+ waitlisted patients, a minimum of 425 to 850 individuals have hereditary conditions where consanguineous relatives share genetic risk and may be medically unsuitable as donors. These patients are simultaneously prohibited from receiving a kidney from genetically unaffected relatives by marriage. For Alport syndrome and Fabry disease—which are X-linked and autosomal dominant conditions respectively, in which obligate carriers may have normal or near-normal renal function—unaffected in-law relatives represent medically appropriate donors who are categorically excluded under the current law. Regarding the age threshold, since Taiwan’s Civil Code reduced the age of majority to 18 years in 2023, potential donors aged between 18 and 19 are legally recognized adults, yet cannot donate a kidney despite being eligible to donate a liver segment to the same recipient. Precise registry data attributing foregone transplants to these restrictions are not currently reported by the Taiwan Organ-Sharing Registry and Patient Promotion Center ; we recommend that the Taiwan Organ-Sharing Registry and Patient Promotion Center prospectively capture donor eligibility barriers as a quality indicator to enable future quantification. Nonetheless, the minimum of 425 to 850 patients with hereditary kidney disease on the current waiting list—many of whom have no eligible consanguineous donor by definition of their genetic diagnosis—represent a policy-attributable gap that harmonization would directly address.

### International Experience

International data demonstrate that expanding donor eligibility with appropriate safeguards increases transplantation without compromising safety. In the United States, approximately 20% of living kidney transplants involve nonrelated donors.^5^ The UK’s Living Kidney Sharing Scheme effectively expands opportunities through national paired exchange.^6^ Germany permits donation between individuals with close emotional relationships through independent evaluation. Israel’s 2008 policy combining economic support with priority allocation significantly increased donation rates, though it raised concerns about potential coercion of vulnerable populations.^7^ These experiences underscore the need for policies that expand access while implementing robust safeguards.

### Expert Consensus: 3 Pillars of Reform

In March and April 2025, expert meetings comprising 14 specialists—transplant surgeons, nephrologists, ethicists, legal scholars, and human rights advocates—formulated consensus recommendations as follows:

**Pillar 1—Expand eligibility:** Extend kidney donor eligibility to relatives by marriage within 5 degrees, consistent with liver donation. This resolves regulatory inconsistency and provides relief for patients with hereditary kidney disease.

**Pillar 2—Unify standards:** Reduce minimum donor age from 20 to 18 years, aligning with liver donation and civil law, respecting adult autonomous decision-making.

**Pillar 3—Strengthen safeguards:** Implement enhanced independent ethics review and comprehensive psychosocial evaluation for all donors, with particular attention to coercion risks for relatives by marriage, including daughters-in-law and foreign spouses (Figure 1).

**Figure 1:**
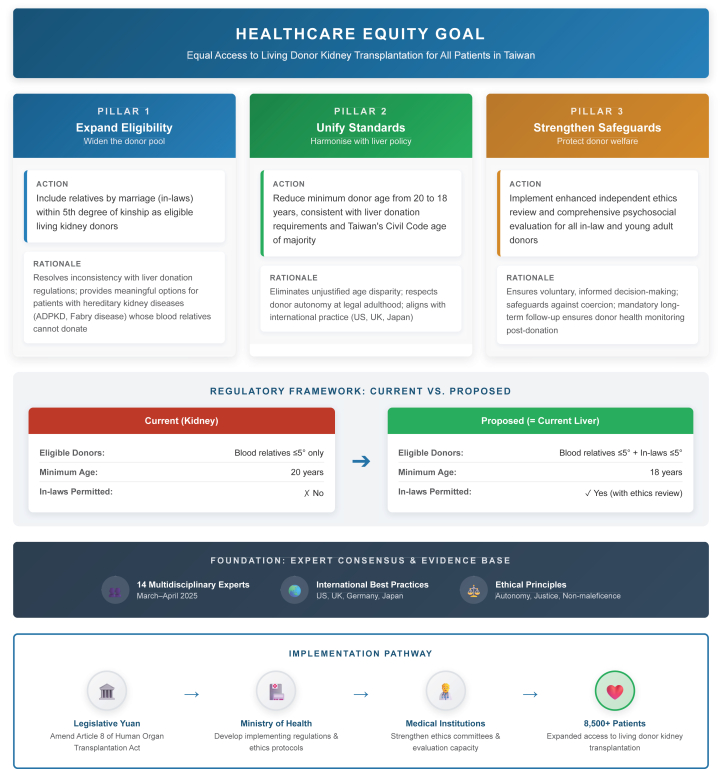
Three Pillars Framework for Living Kidney Donation Policy Reform in Taiwan. This figure illustrates the proposed framework for harmonizing Taiwan's living kidney donation regulations with existing liver donation policy to achieve healthcare equity. **Pillar 1 (Expand Eligibility)** extends donor eligibility to include relatives by marriage within 5 degrees of kinship, addressing the needs of patients with hereditary kidney diseases whose blood relatives cannot donate. **Pillar 2 (Unify Standards)** reduces the minimum donor age from 20 to 18 years, aligning with civil law and liver donation requirements. **Pillar 3 (Strengthen Safeguards)** implements enhanced independent ethics review and comprehensive psychosocial evaluation to protect vulnerable populations. The comparison panel highlights the current regulatory disparity between kidney and liver donation. The framework is based on expert consensus from 14 multidisciplinary specialists (March–April 2025) and international best practices. Implementation requires coordinated legislative, regulatory, and institutional action to expand access for over 8500 patients awaiting kidney transplantation. ADPKD, autosomal dominant polycystic kidney disease; 5°, fifth degree of kinship.

### Protecting Vulnerable Populations

Gender equality and human rights concerns were central to deliberations. Women are disproportionately represented among living donors globally, and cultural expectations may pressure vulnerable individuals.^8^ The expert panel emphasized that reform must include the following: ethics committees with gender equality expertise; psychosocial evaluation specifically screening for coercion; confidential opportunities for donors to decline without family knowledge; and comprehensive informed consent addressing long-term implications.

### Implementation Path

Reforms require coordinated action in the following ways: the Legislative Yuan should prioritize amending Article 8 of the Human Organ Transplantation Act; the Ministry of Health and Welfare should convene stakeholders to develop implementing regulations; and medical institutions should strengthen ethics committee capacity. Future considerations include national paired kidney exchange programs and nondirected donation pathways.

## Conclusion

Taiwan has demonstrated world-leading expertise in living liver transplantation with rigorous ethical oversight. There is no medical or ethical justification for denying kidney transplant candidates the same policy framework. The current regulatory inconsistency represents an unjustified barrier to healthcare equity. Harmonizing kidney donation policy with the successful liver donation framework—while strengthening donor protections—would advance access to superior transplantation outcomes for the over 8500 patients currently waiting.
